# Intramuscular transplantation of bone marrow cells prolongs the lifespan of SOD1^G93A^ mice and modulates expression of prognosis biomarkers of the disease

**DOI:** 10.1186/s13287-018-0843-z

**Published:** 2018-04-06

**Authors:** Amaya Rando, Diego Pastor, Mari Carmen Viso-León, Anna Martínez, Raquel Manzano, Xavier Navarro, Rosario Osta, Salvador Martínez

**Affiliations:** 1 LAGENBIO-I3A, Facultad de Veterinaria, IIS Aragón, Universidad de Zaragoza, Zaragoza, Spain; 2 Centro de Investigación Deporte, Universidad Miguel Hernández de Elche, Alicante, Spain; 3 Instituto de Neurociencias de Alicante, UMH-CSIC, Universidad Miguel Hernández de Elche, Alicante, Spain; 4 Grupo de Neuroplasticidad y Regeneración, Instituto de Neurociencias y Departamento de Biología Celular, Fisiología e Inmunología, Universidad Autónoma de Barcelona, Barcelona, Spain; 5 Department of Physiology, Anatomy and Genetics, University of Oxford, Oxford, UK

## Abstract

**Background:**

Amyotrophic lateral sclerosis (ALS) is a neurodegenerative disorder characterized by progressive muscle weakness, paralysis and death. There is no effective treatment for ALS and stem cell therapy has arisen as a potential therapeutic approach.

**Methods:**

SOD1 mutant mice were used to study the potential neurotrophic effect of bone marrow cells grafted into *quadriceps femoris* muscle.

**Results:**

Bone marrow intramuscular transplants resulted in increased longevity with improved motor function and decreased motoneuron degeneration in the spinal cord. Moreover, the increment of the glial-derived neurotrophic factor and neurotrophin 4 observed in the grafted muscles suggests that this partial neuroprotective effect is mediated by neurotrophic factor release at the neuromuscular junction level. Finally, certain neurodegeneration and muscle disease-specific markers, which are altered in the SOD1^G93A^ mutant mouse and may serve as molecular biomarkers for the early detection of ALS in patients, have been studied with encouraging results.

**Conclusions:**

This work demonstrates that stem cell transplantation in the muscle prolonged the lifespan, increased motoneuron survival and slowed disease progression, which was also assessed by genetic expression analysis.

## Background

Amyotrophic lateral sclerosis (ALS) is the most frequent adult-onset motoneuron degenerative disease, characterized by degeneration of upper and lower motoneurons, which leads to progressive paralysis and death from respiratory failure within 3–5 years of symptom onset [1–3]. The ALS prevalence is 4-6 per 100,000 people [4] and approximately 90% of ALS cases are sporadic (SALS) while the remaining 10% are generally inherited, known as familial ALS (FALS) [5]. The pathogenesis remains unclear but susceptibility to FALS is associated with mutations in various genes, like *TARDBP*, *FUS*, *OPTN*, *VCP*, *UBQLN2*, *C9orf72*, *TBK1* and the most common *SOD1* [6], for a total amount of 20 genes [7]. These genetics causes have allowed creation of transgenic mouse models of FALS. These animal models develop pathological and clinical features closely resembling human ALS, the most frequently used ALS model being a transgenic mouse overexpressing human SOD1 with a G93A mutation (SOD1^G93A^) [8, 9]. Because familial and sporadic ALS share clinical and pathological signs, SOD1^G93A^ mice provide a good model to investigate the pathogenesis of ALS and to test a wide range of potential therapeutic molecules and approaches [10].

There are currently no efficient treatments for this fatal disease, with riluzole being the only medication prescribed, although with slight results [11, 12]. Because of this, different experimental therapies have been tested [13, 14] and among all of them cell therapy has been raised as a promising approach for treating ALS [15, 16]. Different types of stem cells and ways of administration have been tested in experimental models of ALS, and based on these results [17] clinical trials have been conducted with slight but promising outcomes [18–22].

As ALS is a distal axonopathy [23, 24] in which neuromuscular degeneration precedes the onset of clinical symptoms and motor neuron (MN) death [25], some studies have changed their target from the spinal cord to the skeletal muscle, to protect the neuromuscular junctions (NMJs) and reduce MN degeneration by retrograde neurotrophism through axonal projections. In this sense, different strategies such as gene or cell therapy have been used to deliver growth factors into skeletal muscles of animal models of ALS [26, 27]. This type of approach may be considered more feasible due to the accessibility of skeletal muscle and more efficient, where both the MNs and NMJs are protected, preserving the function of the treated muscle [28]. Moreover, skeletal muscle is an accessible tissue that has a direct connection with the nervous system and plays an important role in ALS pathophysiology [29, 30]; therefore, it is possible to carry out studies in this tissue to find molecular markers that could help in establishing diagnosis and prognosis. In a recent study, Calvo et al. [31, 32] showed that different degenerative biomarkers and genes involved in muscle metabolism, maintenance and regeneration are altered in skeletal muscle of SOD1^G93A^ mice, and may serve as genetic biomarkers for monitoring disease progression after experimental therapies.

In the present study we evaluate the efficacy and feasibility of intramuscular transplantation of total bone marrow cells (BMCs) in SOD1^G93A^ mice. BMC grafts prolonged survival, ameliorated MN degeneration and slowed the clinical course of the disease. In parallel, a downregulation in the expression of genetic biomarkers also demonstrated the therapeutic benefit of BMC grafts. We hypothesized that BMCs increased the bioavailability of the neurotrophic factors glial-derived neurotrophic factor (GDNF) and neurotrophin 4 (NT4) in the skeletal muscle, preserving the integrity of the NMJs.

## Methods

### Animal care

All experimental procedures were approved by the Ethics Committee of the University of Zaragoza and followed the international (Directive 2010/63/EU) and national (RD 53/2010) guidelines for the use of laboratory animals. Transgenic B6SJLTg(SOD1-G93A)1Gur/J mice expressing a high copy number of the G93A mutant form of human SOD1 (SOD1^G93A^) [8] and the green fluorescent protein (GFP) (C57Bl/6-Tg(ACTB-EGFP)1Osb/J) were housed under a 12-h light/12-h dark cycle at 21–23 °C with a relative humidity of 55% in the animal facilities of the institution. Food and water were available ad libitum. When necessary, euthanasia was performed by CO_2_ inhalation and anesthesia was induced by isoflurane inhalation.

### Locomotor behavioral assays

At 70 days of age, balanced male and female SOD1^G93A^ mice were treated blindly by injecting in both hind limbs either BMCs (*n* = 20) or fresh medium (*n* = 20). The onset and progression of the disease was analyzed using rotarod and treadmill tests. Mice were trained 2 weeks before injection, allowed 1 week to recover and then the tests were performed weekly [33].

The rotarod test was performed on an 8500 Rotarod (Leica Scientific Instruments). The time an animal could remain on the rotating cylinder which uniformly increased speed from 4 to 40 rpm over a 5-min period was measured. Animals were tested eight times in each session and the best performance was recorded.

For the treadmill test, the LE 8700 model was used (Leica Scientific Instrument). The runway, when in movement, pushes the animal to the shock grid, set at 0.4 mA. The treadmill is uniformly accelerated until the mouse reaches the shock grid, which corresponds with the maximum speed the mouse can attain. Each mouse was placed in the treadmill eight times per session and the maximum speed obtained was recorded. With this assay it is possible to measure the maximum speed attained by mouse disease models, including neurodegenerative models [34].

### Lifespan analysis method

For the lifespan study, mice were sacrificed when they were unable to right themselves within 30 s after being placed on their side; this point was considered as the survival endpoint according to the guidelines for preclinical testing and colony management [35, 36].

### Separation method of BMCs from GFP mice

Femurs were dissected from GFP-positive mice 6–8 weeks old, sacrificed by cervical dislocation. Bone marrow was extracted by pressure with a 30G syringe, and single-cell suspensions were obtained by mechanical dissociation. The cells were then counted using a Neubauer camera and resuspended in fresh medium (D-MEM; Invitrogen) at the adequate concentration.

### Cell transplantation

Cell transplantation was performed as described previously [37]. Briefly, bone marrow cells were isolated from GFP mice and immediately 10 μl of medium containing 300,000 BMCs was injected into 70-day-old SOD1^G93A^ q*uadriceps femoris*. After visualizing the muscle, the cells were inoculated at two different points of inoculation. A negative control group was similarly injected with 10 μl of fresh medium (D-MEM; Invitrogen). SOD1^G93A^ mice transplanted with total BMCs obtained from GFP mice were designated BMC-transplanted mice and those injected with culture medium were designated sham-injected mice.

### Tissue preparation and immunohistofluorescence

Five weeks after injection, the mice were anesthetized and then perfused with 4% paraformaldehyde in phosphate buffered saline (PBS). For immunofluorescence, spinal cords and q*uadriceps femoris* muscle were cryopreserved and embedded in tissue freezing medium and quickly frozen (*n* = 6). Then, transverse spinal cord sections (20 μm) and longitudinal muscle section (20 μm) were obtained serially with a cryostat. Skeletal muscle inmunofluorescence was performed to detect the grafted cells as described previously [37]. Histological samples were observed using a fluorescence microscope (Leica DMR; Leica Microsystems) and micrographs were taken with a confocal microscope (Leica DMR).

### Motoneuron counting

At 70 days, transplanted mice received total BMCs in one of their hind limbs and cell culture medium in the other. Sham-injected mice received cell culture medium in both hind limbs. Four weeks later, 10 μl of 1,1′-dioctadecyl-3,3,3′,3′-tetramethylindocarbocyanineperchlorate (DiI) dissolved in 80% ethanol was injected into both *quadriceps femoris*. After a survival period of 5 days, which is required for complete retrograde MN labeling, spinal cord sections from L1 to S2 were checked every 100 μm for the number of stained neurons. Only neurons located in the ventral horn that were DiI-positive and presented a distinct nucleus were counted. Stained neurons from the right and left ventral horns were counted separately and compared between BMCs of transplanted and sham mice. Results were expressed as total number of MNs stained in the selected sections.

### Gene expression

At the age of 120 days, q*uadriceps femoris* muscles from mice grafted with BMCs in both hind limbs (*n* = 6) and from sham-injected mice (*n* = 6) were dissected and immediately frozen in liquid nitrogen. Each muscle was pulverized using a Frozen Cell Crasher and half of the power was kept for protein extraction. Prior to being processed according to the TRIzol Reagent protocol (Invitrogen) for RNA extraction, powered muscle was further homogenized using a PRO200 homogenizer (PRO Scientific Inc.). Genomic DNA was eliminated using the Turbo DNA-free Kit (Ambion) and cDNA was synthesized from 1 μg for RNA using the Superscript II First Strand kit (Invitrogen), all according to the manufacturer’s instructions. The presence of engrafted GFP cells in muscle tissue was confirmed by reverse transcriptase PCR (RT-PCR). RT-PCR was performed as described previously [38]. Quantitative real-time PCR (qRT-PCR) for ALS biomarkers was performed using TaqMan^®^ primer/probe mixtures. Reactions were run using the StepOne Plus Real-Time PCR System (Applied Biosystems) according to the manufacturer’s instructions. For neurotrophic factors, qRT-PCR was performed using Power SYBR Green Master mix (Applied Biosystems). The primers [39] and TaqMan^®^ primer/probe mixtures used are presented in Tables 1 and 2. *Glyceraldehyde-3-phosphate dehydrogenase* (*GAPDH)* and *beta actin* (*β-actin)* were used for normalization [40] and relative gene expression compared with sham-injected mice was determined using the 2^–ΔΔCT^ method [41].

**Table 1:** Primer sequences used for gene expression analysis

Gene	5′ → 3′	Primer sequence
*NT4*	Forward	TGAGCTGGCAGTATGCGAC
	Reverse	CAGCGCGTCTCGAAGAAGT
*NGF*	Forward	GCACTACACCCATCAAGTTCA
	Reverse	TCCTGAGTCATGCTCACAAGT
*BDNF*	Forward	TCATACTTCGGTTGCATGAAGG
	Reverse	GTCCGTGGACGTTTACTTCTTT
*NT3*	Forward	AGTTTGCCGGAAGACTCTCTC
	Reverse	GGGTGCTCTGGTAATTTTCCTTA
*GDNF*	Forward	CGCCGGTAAGAGGCTTCTC
	Reverse	CGTCATCAAACTGGTCAGGATAA
*VEGF*	Forward	GCCAGACAGGGTTGCCATAC
	Reverse	GGAGTGGGATGGATGATGTCAG
*IGF1*	Forward	CTGGACCAGAGACCCTTTGC
	Reverse	GGACGGGGACTTCTGAGTCTT
*EGF*	Forward	AGCATCTCTCGGATTGACCCA
	Reverse	CCTGTCCCGTTAAGGAAAACTCT
*GAPDH*	Forward	AGGTCGGTGTGAACGGATTTG
	Reverse	GGGGTCGTTGATGGCAACA
*β-actin*	Forward	AGAGGGAAATCGTGCGTGAC
	Reverse	CAATAGTGATGACCTGGCCGT
*GFP*	Forward	CTG CTG CCC GAC AAC CA
	Reverse	GAA CTC CAG CAG GAC GAC CAT GTG

**Table 2:** TaqMan^®^ probe and primer mixtures used in gene expression assays

Gene	Part number
*Ankrd1*	Mm00496512_m1
*Calm*	Mm00486655_m1
*Col19a1*	Mm00483576_m1
*Gsr*	Mm00833903_m1
*Impa1*	Mm00497770_m1
*Mt2*	Mm00809556_s1
*Mef2c*	Mm00600423_m1
*Myod1*	Mm00440387_m1
*Myf5*	Mm00435125_m1
*Myog*	Mm00446194_m1
*Nnt*	Mm00435154_m1
*Pax7*	Mm00834079_m1
*Rrad*	Mm00451053_m1
*Rtn4*	Mm00445861_m1
*Sln*	Mm00481536_m1
*Snx10*	Mm00511049_m1
*β-actin*	4352933E
*GAPDH*	4352932E

### Western blot assay

Powdered tissue was homogenized in RIPA lyses buffer with protease inhibitors (Complete; Roche); the homogenate was centrifuged at 10,000 × *g* for 10 min at 4 °C and the resulting supernatants were collected. Next, the protein concentration was determined by BCA method (Sigma Aldrich). Then, 40 μg of total protein was subjected to SDS/PAGE and transferred to PVDF membranes (Amersham Biosciences). For inmunodetection, membranes were incubated in blocking solution (5% nonfat milk) overnight at 4 °C and then incubated for 1 h with primary antibodies against NT-4 (1:500; Santa Cruz), brain-derived neurotrophic factor (BDNF) (1:500; Santa Cruz), GDNF (1:500; Santa Cruz) and GAPDH (1:1000; Santa Cruz). After incubation with HRP-conjugated secondary antibodies (Santa Cruz), bands were visualized by enhanced chemiluminescent reagent (GE Healthcare Life Science). Immunoblots were exposed and scanned, and, finally, densitometry was measured with AlphaEaseFC software (Bonsai).

### Statistical analysis

Data are expressed as mean ± SEM. Statistical analysis for behavioral assays was performed by means of one-tailed Student’s *t* test; for both protein and gene expression quantification, two-tailed Student’s *t* test was used. Nonparametric Mann–Whitney test was used for cross-sectional areas. Kaplan–Meier survival curves and the Mantel–Cox log-rank test were used to analyze disease onset and lifespan. Values were considered statistically significant at *p* < 0.05. Tendency was assumed when 0.1 > *p* > 0.5.

## Results

### Bone marrow transplant improved evolution and prolonged survival of SOD1G93A mice and downregulated the expression of genetic markers of ALS

#### Immunofluorescence and RT-PCR revealed the presence of GFP-positive cells in the skeletal muscle of SOD1^G93A^ mice 35 days after transplantation

**Fig. 1:**
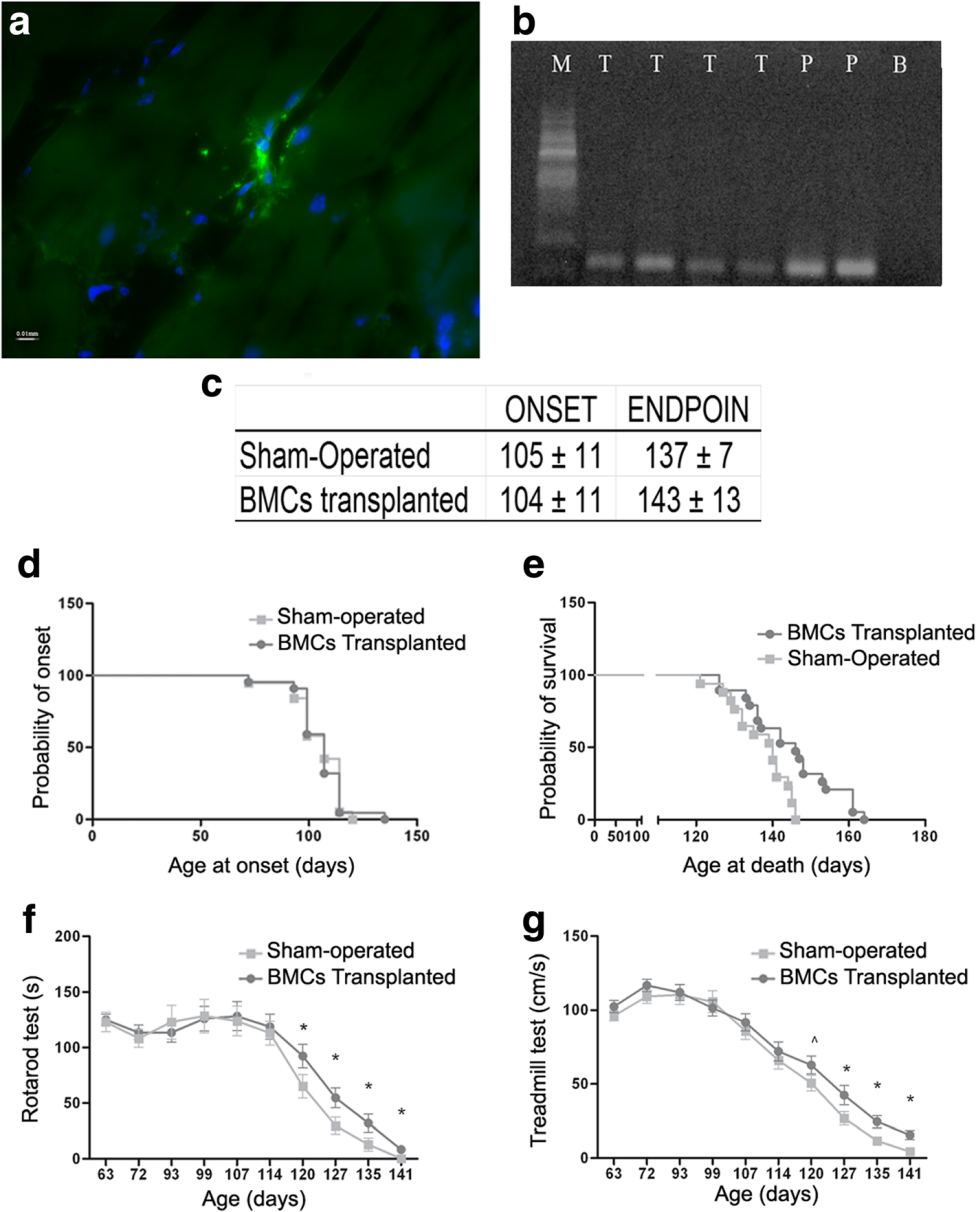
Effect of BMC transplantation into skeletal muscle of ALS transgenic SOD1G93A mice. **a** Representative longitudinal section of *quadriceps femoris* muscle showing immunohistochemical labeling for GFP cells (green). Nuclei stained with DAPI (blue). **b** Effect of BMC transplant on course of disease in SOD1^G93A^ mice. BMC-treated mice are shown in dark gray and sham-injected mice in light gray. RT-PCR amplification for the detection of GFP expression in mouse *quadriceps femoris* after intramuscular BMC transplantation (T treated mice, P GFP cells used as positive control, B Black control, M RNA Marker). **c** Onset of symptoms and mortality of BMC-treated and sham-injected mice presented by days. **d**, **e** Cumulative probability of **d** onset of disease symptoms and **e** survival. **f**, **g** Motor functions assessed by rotarod and treadmill tests. **f** Latency of fall when submitted to rotarod test. **g** Maximum speed attained as measured on the treadmill. *n* = 20 sex-balanced animals per group. ^*p* < 0.10; *p < 0.05. Error bars indicate SEM. BMC bone marrow cell

We first assessed whether the transplanted cells were able to survive in the host skeletal muscle. To this aim, q*uadriceps femoris* muscle was tested for the presence of GFP-positive cells. Five weeks after transplantation, immunofluorescence against GFP demonstrated the presence of GFP-positive cells in BMC-transplanted mice (Fig. 1a). Furthermore, RT-PCR also revealed the expression of GFP in the q*uadriceps femoris* of BMC-transplanted mice but not in the muscle of the sham-injected mice (Fig. 1b). From the microphotography it can be observed that GFP-positive cells remain in the skeletal muscle 35 days after transplantation, but in a low concentration; in fact, it was difficult to find them, probably produced by cell death after transplant, as Gubert et al. [42] found in other tissue. The image corresponds to a longitudinal section close to one of the inoculation points as the cells are not able to migrate through the tissue.

#### Bone marrow grafts significantly improved disease clinical outcomes and prolonged survival of SOD1^G93A^ mice

BMC transplants did not significantly delay disease onset as compared to the sham-injected group (Fig. 1c, d). However, the duration of symptomatic phase was prolonged in the BMC-transplanted group (Fig. 1c, e). Regarding the lifespan, the BMC-transplanted mice showed significantly longer survival rates compared to the sham-injected mice (143 ± 13 and 137 ± 7 days respectively; log-rank test, *p* < 0.005) (Fig. 1c, e). The beneficial effect of BMC transplants on motor function and coordination was assessed by rotarod and treadmill tests. Concerning the rotarod test, BMC transplants did not delay the appearance of the first signs of motor deficiency. However, the average rotarod performance of the BMC-transplanted mice from 120 to 140 days was significantly improved (*p* < 0.05) (Fig. 1f). Similarly, from 110 to 140 days, the BMC-transplanted mice performed better in the treadmill test than did the sham-injected mice (Fig. 1g).

#### Bone marrow grafts induced downregulation of genetic biomarkers of ALS

**Fig. 2:**
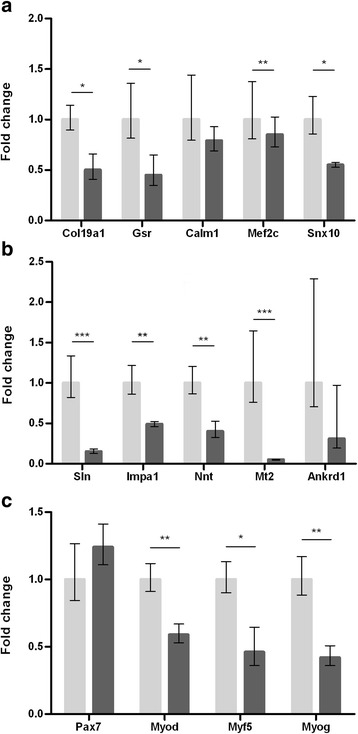
Quantification of transcript levels of genes described to be significantly upregulated in skeletal muscle of SOD1G93A mice at late phases of the disease (120 days). **a** Fold-change in expression of *Col19a1*, *Gsr*, *Snx10*, *Calm1* and *Mef2c.* **b** Downregulation of *Sln*, *Impa1*, *Nnt*, *Mt2* and *Ankrd1.* **c** Transcript levels of *Pax7* and myogenic regulatory factors (*Myod*, *Myf5*, *Myog*). Results shown as fold-change in BMC-treated SOD1^G93A^ mice muscles (dark gray bars) relative to sham-injected muscles (light gray bars). *p < 0.05; **p < 0.01; ***p < 0.001

The transcript levels of five potential ALS longevity biomarkers recently described by our group were quantified [31] (Fig. 2a). Specifically, transcripts of *collagen, type XIX, alpha 1* (*Col19a1), glutathione reductase (Gsr)* and *sorting nexin 10* (*Snx10)* were significantly reduced in the BMC transplant mice (Fig. 2a) (*p* < 0.05). As the expression of these genes negatively correlates to longevity, the downregulation observed agrees with the lifespan extension in BMC-transplanted mice. Surprisingly, no differences were found in *Calmodulin 1* (*Calm1)* and *myocyte enhancer factor 2C* (*Mef2c)* transcripts, involved in muscle damage and myogenic pathways respectively (Fig. 2a).

Subsequently, we analyzed the expression profile of several genes involved in different pathways of muscle metabolism and structure maintenance that have been described to be significantly upregulated in skeletal muscle of SOD1^G93A^ at late symptomatic phases of the disease [31]. The transcriptional level of *sarcolipin* (*Sln)*, involved in calcium homeostasis, showed a robust downregulation (*p* < 0.001) (Fig. 2b). Three genes related to metabolic processes, *inositol(myo)-1(or 4)-monophosphatase 1 (Impa1), mitochondrial nicotinamide nucleotide transhydrogenase (Nnt)* and *metallothionein 2 (Mt2),* were strongly downregulated as well, this reduction being especially remarkable in the case of *Mt2* (Student’s *t* test: *Impa1 and Nnt*, *p* < 0.01; *Mt2, p* < 0.001) (Fig. 2b). Surprisingly, the expression of *Ankyrin repeat domain 1* (*Ankrd1*), a muscle plasticity and injury marker [43], was not modified. Although a tendency for downregulation can be appreciated in the graph, it was not statistically significant due to a large intragroup variability (Fig. 2b). Previous studies have demonstrated that transcriptional levels of *paired box 7 (Pax7)* and myogenic regulatory factors *myogenic differentiation 1* (*Myod1), myogenic factor 5 (Myf5)* and *myogenin* (*Myog)* are dramatically upregulated in the muscle of SOD1^G93A^ mice at a late symptomatic stage of the disease (120 days) compared with aged wild-type littermates [44]. We analyzed *Pax7* and MRF transcripts and detected a significant and strong reduction in the RNA level of MRFs in the BMC-transplanted mice (Fig. 2c) (*p* < 0.05 for *Myf5* and *p* < 0.01 for *Myod* and *Myog*). However, no differences were observed in *Pax7* expression (Fig. 2c).

Overall, these results would evidence muscle metabolic restoration after muscle BMC engraftment in the SOD1^G93A^ mice model—the mechanism underlying the observed motor function improvement and prolonged lifespan of the mice.

### BMC graft improved maintenance of NMJs and increased survival of motoneurons in the spinal cord in SOD1G93A mice, and these effects are mediated by neurotrophic factor release

#### *BMC transplant increased the survival of motoneurons in the spinal cord of SOD1*^*G93A*^*mice*

**Fig. 3:**
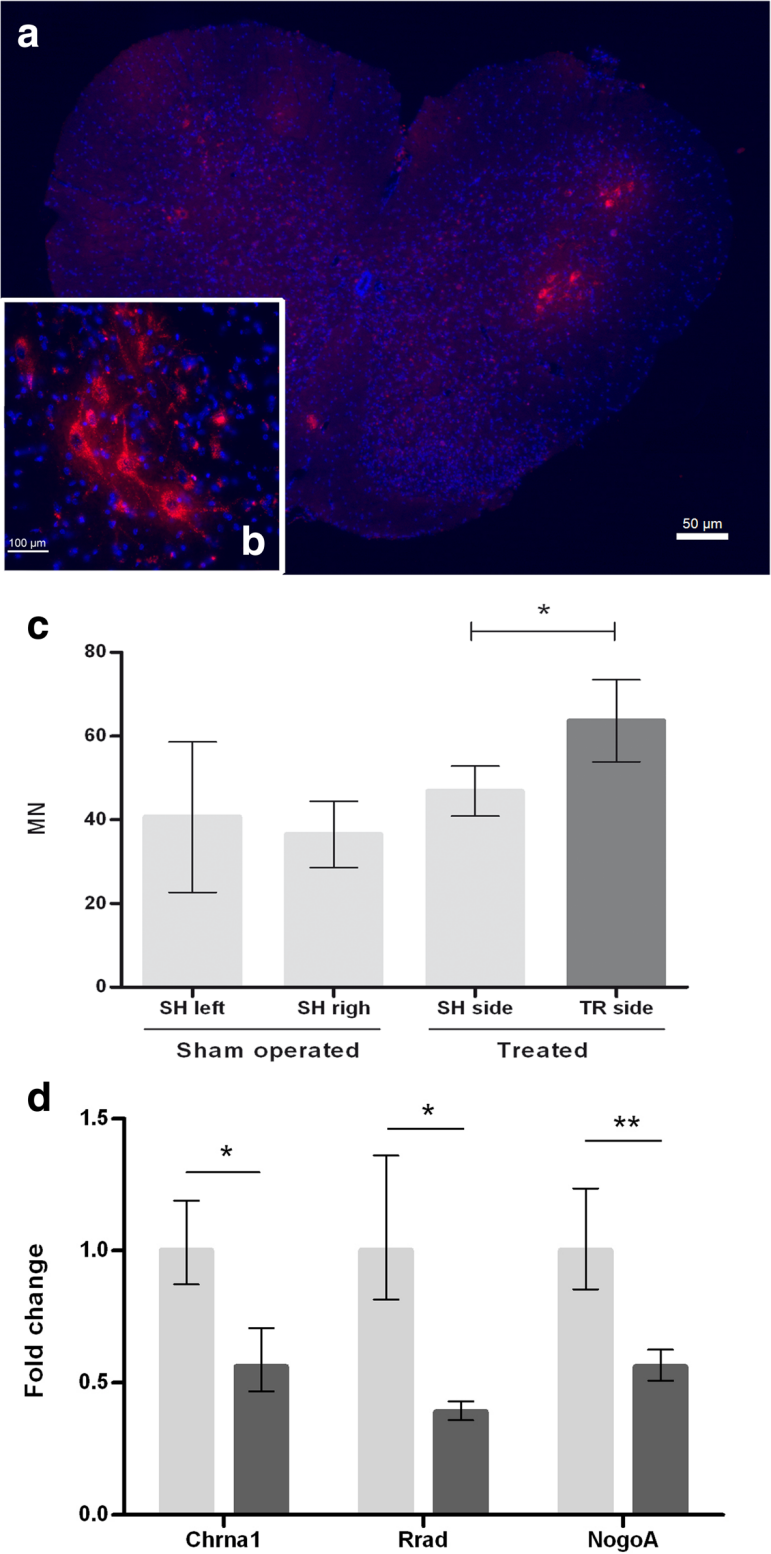
Spinal cord motoneuron (MN) counting and neuromuscular state after BMC transplantation (at 120 days). **a** DiI-labeled MNs (red) in the spinal cord, nuclei stained with DAPI (blue). **b** 40× image of DiI-stained MN. **c** Right: number of MNs in mice treated with bone marrow on one hind limb versus the contralateral limb. *p < 0.05. Error bars indicate SEM. *n* = 6 mice. Left: number of MNs in the spinal cord of sham-injected mice. Error bars indicate SEM. *n* = 5. **d** *Chrna1*, *Rrad* and *NogoA* transcript levels in BMC-treated mice relative to sham-injected mice. SH = Sham-operated; TR = Treated. *p < 0.05; **p < 0.01

DiI was injected into both *quadriceps femoris* of BMC-transplanted and sham-injected mice, and after 5 days the mice were perfused and spinal cords extracted for immunohistochemical analysis. Since DiI is transported retrogradely from the muscle to the body of the MN, only the surviving MN that still innervated those muscles was stained and considered for quantification (Fig. 3a). In the case of BMC-transplanted animals, the left ventral horn, linked with the treated limb, presented a higher number of retrogradely labeled MN compared to the right ventral horn, corresponding to the nontreated limb (right, treated side 63.66 ± 9.75; left, contralateral side 45 ± 5.95; *p* < 0.05) (Fig. 3c). In the sham-injected mice, no significant differences were found in the number of stained MNs between the right and left sides of the spinal cords (SH left 40.67 ± 17.95, SH right 36.5 ± 7.95; *p* = 0.83) (Fig. 3c). These results indicate that the mice treated in one limb with BMCs presented a higher number of surviving MNs innervating that limb compared to the contralateral side. Interestingly, fluorescent cellular debris was abundant in the nontransplanted limb, in some cases around the remaining innervating MNs (Fig. 3a), indicating that retrograded labeled MNs have suffered cell death during the 5 days after DiI labeling. In contrast, almost no fluorescent cellular debris was observed in the transplanted side, suggesting a less active degenerative process.

#### BMC grafts improved maintenance of NMJs

To determine the effect of BMC grafts on the NMJs, we quantified transcript levels of *Ras-related associated with diabetes* (*Rrad*), *cholinergic receptor, nicotinic, alpha 1* (*Chrna1*) and *Reticulon-4* (*NogoA*), a well-known inhibitor of axonal regeneration and promoter of NMJ destruction [30, 45]. *Rrad* is an early marker of muscle injury, correlating its expression positively with ALS both in patients and animal models from the presymptomatic stages of the disease [40, 46]. Similarly, the denervation rate was assessed by *Chrma1* transcript levels, as the expression of its receptor is modulated by electrical activity [47–49]. In 120-day-old SOD1^G93A^ mice, *Rrad*, *Chrna1* and *NogoA* transcripts exhibited a considerable reduction in the BMC-transplanted group (Fig. 3d). These observations supported the idea that BMC transplant improves NMJ stability and may lie behind the observed motor function improvement and prolonged lifespan of the mice.

### The therapeutic effect of BMC grafts was mediated by neurotrophic factor release

**Fig. 4:**
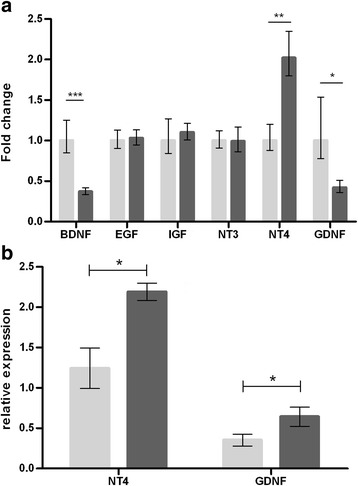
Gene and protein expression of neurotrophic factors. **a** qRT-PCR analysis of *EGF, IGF, NT3, NT4* and *GDNF* transcript levels in BMC-treated mice (dark gray) compared to sham-injected mice (light gray). *p < 0.05; **p < 0.01; ***p < 0.001. Error bars indicate SEM. *n* = 6. **b** Fold-changes in expression of GDNF and NT4 proteins in skeletal muscle of BMC-treated mice (dark gray) and sham-injected mice (light gray) assessed by western blot analysis. Quantities shown as ratios to GAPDH. *p < 0.05. Error bars indicate SEM. *n* = 6 per group. No differences observed in *IGF*, *NT3* and *VEGF* expression between both groups, and no *NGF* detected (data not shown). However, there was a solid decrease in *GDNF* (*p* < 0.05) and a strong upregulation of *NT4* (*p* < 0.01) in BMC-treated mice. BDNF brain-derived neurotrophic factor, EGF *epidermal growth factor, IGF Insulin-like growth factor, NT neurotrophin, GDNF g*lial-derived neurotrophic factor

Mesenchymal stem cells from the bone marrow are known to secrete different trophic factors involved in neuroprotection [50]. Therefore, the transcription levels of *GNDF*, *epidermal growth factor (EGF), vascular endothelial growth factor (VEGF), insulin-like growth factor (IGF), nerve growth factor (NGF), neurotrophin 3 (NT3)* and *NT4* were analyzed in the BMC-grafted muscles compared to sham-injected muscles (Fig. 4a).

Consistently, NT4 and GDNF protein levels showed a 2-fold increase (Fig. 4b). NT4 is produced by MNs and muscle fibers, and its release by skeletal muscle is positively regulated by muscle activity [51]. GDNF is a well-known neurotrophic factor that has been proposed as a therapeutic agent for ALS with promising results [52].

These results support the hypothesis that the therapeutic effect of BM grafts is localized at the NMJs and mediated, at least in part, by the neurotrophic factors NT4 and GDNF through the induction of axonal sprouting and continuous synaptic remodeling of NMJs, thus enhancing neuromuscular transmission [53–55].

## Discussion

Here, we demonstrated that the intramuscular transplant of total BMCs presented some improvements in the pathology course, prolonging the lifespan and ameliorating the disease phenotype of SOD1^G93A^ mice, a suitable, well-known and deeply characterized animal model of ALS [35, 56].

Given the lack of effective treatment for ALS patients, a wide variety of experimental therapeutic strategies have arisen including transplantation with mesenchymal stem cells from different origins such as adipose tissue and bone marrow [57–60], as well as umbilical cord blood and neural stem cells [61–64]. Traditionally, these studies have been focused on the capability of the grafted cells to reach and exert their beneficial effects in the central nervous system by differentiating into nerve cells or releasing neurotrophic factors that could improve the survival of MNs, and for these reasons, intracerebroventricular, intrathecal, intravenous and intraspinal administration have been the most frequent delivery methods employed (reviewed in [17]).

However, despite ALS being considered to have a distal axonopathy component in which MN degeneration occurs as a dying back disorder [23, 25, 65], and muscle disturbance being an early event in the disease that has even been considered a primary target in ALS pathogenesis [29, 30, 66], to our knowledge skeletal muscle has been barely proposed as a target organ [27, 37, 52] and the effects of stem cell engraftment on muscles have been poorly studied in animal models of neuromuscular diseases and in particular in ALS. Previous results from Pastor et al. [37] showed that BMC grafts into *quadriceps femoris* muscle were capable of improving the survival of MNs in the spinal cord and the motor functions of mdf/ocd mice, a motoneuron degenerative mouse model, through the production and secretion of trophic factors such as GDNF. Here we employed the SOD1^G93A^ mouse, a more suitable murine model for ALS [35], carried out survival studies and, remarkably, assessed the efficiency of BMC transplant by analyzing the expression of genetic biomarkers described previously by our group. Total BMCs constitute a heterogeneous population which includes both mature and immature hematopoietic cells and mesenchymal stem cells (MSCs) [67]. BMCs can be relatively easily and painlessly isolated and require minimum manipulation prior to transplant, which has facilitated their translation into clinical trials for ALS [18] and other neurological diseases, demonstrating that this approach is feasible and safe [68]. Thinking in a likely future translation, we simultaneously performed BMC extraction and transplant following a protocol that reduces the manipulation of the cells to a minimum and highly resembles the clinical procedure used in previous clinical trials. In addition to technical reasons, in this study BMCs were chosen because we had observed previously that the therapeutic effect was higher when transplanting total BMCs instead of MSCs alone, probably due to a synergic effect between the different bone marrow populations and an increment in the survival rate of the grafted cells [33]. Other cells from BMCs like monocytes or macrophages should be related to the treatment effect. ALS patient macrophages can contribute to MN loss and inflammatory response is increased in muscles [69]; on the other side, a graft of healthy MSCs modified to produce higher volumes of GDNF reduced the local inflammatory process in ALS muscles and improved the NMJ state [70]. The effect of external healthy macrophage graft should be studied in future research to increase our knowledge about its separate possible effect.

Bone marrow mesenchymal stem cells are able to synthetize and secrete neurotrophic factors [50, 71, 72] and have immunomodulatory properties. Although theoretically possible, in this study we did not observe any fusiform GFP cell suggesting muscle differentiation. We found a significant increase in NT4 and GDNF protein levels induced by BMC grafts; and therefore we speculated that BMCs exert their therapeutic effect by releasing neurotrophic factors. In agreement, previous studies showed that BMC transplant from GDNF knock-out embryos had no therapeutic effects [33], so it is reasonable to think that GDNF contributes to the BMC therapeutic effect. However, we cannot rule out the possibility that these cells may modulate the secretion activity of the skeletal muscle toward a neuroprotective phenotype; future studies of gene silencing with siRNA against GDNF and NT4 should be designed to answer this question, and to clarify whether we are showing a direct effect of BMC grafting or a paracrine effect of the BMCs over local tissue. Regardless of the source, both GDNF and NT4 would contribute to skeletal muscle homeostasis and NMJ stabilization. GDNF is important for the maintenance of NMJs and enhanced plasticity and remodeling of NMJs after exogenous administration [55, 73]. In ALS pathology, GDNF could be preventing axonal terminal degeneration and retrograde transportation to MNs. Concerning NT4, in-vitro studies demonstrated that NT4 potently prevented apoptotic MN death [74] in a model of chronic MN degeneration induced by malonate [75] or glutamate [74, 76], and had trophic effects on MNs in vivo [77–79]. These trophic properties lead us to consider NT4 as a potential candidate for the treatment of ALS. In ALS pathogenesis, muscle atrophy is observed at late phases of the disease when the denervation process is largely advanced [8, 80], which led us to focus on the destruction of the NMJs as this occurs as an early pathological event [30]. Therefore, the overexpression of NT4 at mRNA and protein levels in BMC grafted muscles suggest an effect of BMCs in the maintenance and stabilization of active synaptic connections that could initially compensate the denervation and loss of motor function abilities. Supporting this idea, *NT4* mRNA levels decrease after blockade of neuromuscular transmission and increase after electrical stimulation and during postnatal development, suggesting a role in activity-dependent remodeling and maintenance of adult motor innervation and neuromuscular performance [53].

In this sense, recent contributions suggest that MN loss in ALS occurs in a dying back pattern starting from skeletal muscle and NMJ abnormalities and progressing to the neuronal cell body [23, 30, 65, 81]; and skeletal muscle-derived [27, 52, 82] but not motoneuron-derived GDNF expanded the lifespan of SOD1^G93A^ mice [31, 83]. In agreement, the transcript levels of *Rrad*, regulated by oxidative stress [40, 46], *Nogo*-*A*, an axonal regeneration inhibitor [30, 45], and *Chrna1*, upregulated by electrical activity [47–49], showed a consistent downregulation in the transplanted SOD1^G93A^ mice skeletal muscle. These three genes are considered neurodegenerative biomarkers and were progressively upregulated in muscle biopsies from presymptomatic SOD1^G93A^ mice [84]. Surprisingly, we observed a downregulation of *GDNF* mRNA expression that may indicate a rapid translation into protein. Moreover, *GDNF* mRNA levels in muscle increase as a response to ongoing denervation [85] and correlate with the number of partially atrophic muscle fibers [86]. The observed downregulation in the transcript levels of *GDNF* may indicate a decline in the progression of denervation atrophy. In agreement, the expression levels of *GDNF* transcripts in biopsies from ALS patients were significantly increased compared to biopsies from healthy donors [85, 86]. As mentioned previously, diagnosis of ALS is based on clinical and electrophysiological criteria which are difficult to unify [87]. Hence, one of the current challenges for researchers in ALS is to obtain reliable and easily measurable biomarkers that would greatly facilitate early diagnosis and monitoring of disease progression and effectiveness of therapies. Assessments of different parameters in cerebrospinal fluid, muscle or plasma, and electrophysiological measurements or neuroimaging techniques, have been proposed as potential diagnosis or prognosis biomarkers both in animal models and ALS patients [88]. In this sense, Calvo et al. [31] reported a set of five candidate genes (*Col19a1, Gsr, Snx10, Calm1 and Mef2c*) whose expression in skeletal muscle is upregulated and negatively correlates with longevity in the SOD1^G93A^ mouse model. In agreement with the prolonged lifespan of the BMC-transplanted mice, we found downregulation of the expression of *Col19a1, Gsr* and *Snx10* biomarkers. Therefore, the modulation of the expression of these genes in addition to all of the evidence shown supports the idea that BMCs exerted beneficial effects on SOD1^G93A^ mice.

## Conclusions

Overall, here we present an accurate, safe and effective therapeutic approach to rescue the ALS phenotype, based on BMC transplant in the *quadriceps femoris* muscle. Total BMCs transplanted into SOD1^G93A^ mice muscle slowed the clinical course of the disease and prolonged survival when compared to the sham-injected mice, supporting the feasibility and efficacy of this type of cell transplantation as a promising therapeutic strategy for ALS. Moreover, we monitor the effect of the therapy by analyzing the evolution of ALS-specific genetic biomarkers which are useful in the development of preclinical and clinical trials. Finally, we hypothesize that NMJ stabilization by BMCs producing neurotrophic factors GDNF and NT4 may lie beneath the beneficial effects on MN rescue. However, the small number of grafted cells found at the end of the process makes us think that more complex therapies could improve clinical results, with more than one graft, or maybe different grafts for different objectives.

## Abbreviations

ALS: Amyotrophic lateral sclerosis
Ankrd1: Ankyrin repeat domain 1
BM: Bone marrow
BMC: Bone marrow cell
Calm1: Calmodulin 1
Chrna1: Cholinergic receptor, nicotinic, alpha 1
Col19a1: Collagen, type XIX, alpha 1
EGF: Epidermal growth factor
GDNF: Glial-derived neurotrophic factor
GFP: Green fluorescence protein
Gsr: Glutathione reductase
IGF: Insulin-like growth factor
Impa 1: Inositol(myo)-1(or 4)-monophosphatase 1
Mef2c: Myocyte enhancer factor 2C
MN: Motor neuron
mRNA: Messenger RNA
MSC: Mesenchymal stem cell
Mt2: Metallothionein 2
Myf5: Myogenic factor 5
Myod1: Myogenic regulatory factor, myogenic differentiation 1
Myog: Myogenin
NGF: Nerve growth factor
NMJ: Neuromuscular junction
Nnt: Mitochondrial nicotinamide nucleotide transhydrogenase
NogoA: Reticulon-4
NT3: Neurotrophin 3
NT4: Neurotrophin 4
Pax7: Paired box 7
PVDF: Polyvinylidene fluoride
RIPA: Radioimmunoprecipitation assay
Rrad: Ras-related associated with diabetes
RT-PCR: Reverse transcription polymerase chain reaction
SDS/PAGE: Sodium dodecyl sulfate polyacrylamide gel electrophoresis
Sln: Sarcolipin
Snx10: Sorting nexin 10
SOD1: Superoxide dismutase
VEGF: Vascular endothelial growth factor
